# Comparison of scatter and partial volume correction techniques for quantitative SPECT imaging of ^225^Ac

**DOI:** 10.1186/s40658-025-00800-0

**Published:** 2025-10-02

**Authors:** Grigory Liubchenko, Guido Böning, Mikhail Rumiantcev, Adrian J. Zounek, Mathias J. Zacherl, Gabriel Sheikh, Sandra Resch, Rudolf A. Werner, Sibylle I. Ziegler, Astrid Delker

**Affiliations:** 1 Department of Nuclear Medicine, LMU University Hospital, LMU Munich, Marchioninistrasse 15, 81377 Munich, Germany; 2 The Russell H Morgan Department of Radiology and Radiological Sciences, Division of Nuclear Medicine and Molecular Imaging, Johns Hopkins School of Medicine, Baltimore, MD USA; 3 Department of Nuclear Medicine, Hannover Medical School (MHH), Hannover, Germany

**Keywords:** ^225^Ac, SPECT, Quantitative imaging, Partial volume correction, Targeted alpha therapy, Dosimetry

## Abstract

**Background:**

The extreme low-count regime for clinical ^225^Ac-SPECT imaging poses a challenge to energy-window based scatter correction (EWSC) methods. Moreover, SPECT imaging suffers from partial volume effects (PVE), which can degrade quantification and lead to an underestimation of the absorbed dose estimations, especially in small structures such as lesions. The aim of this study was to investigate the impact of scatter correction and partial volume correction (PVC) techniques on post-therapeutic imaging of the three imageable photopeaks of ^225^Ac.

**Methods:**

A phantom with three 3D-printed spheres (191, 100, 48 ml) was imaged to compare transmission-dependent scatter correction (TDSC) to EWSC (440, 218 keV)/no scatter correction (no SC) (78 keV), as well as the impact of iterative Yang (IY)- and Richardson-Lucy (RL)-based PVC techniques, in terms of contrast-to-noise ratios (CNR) and recovery coefficients (RC). These scatter correction and PVC methods were also compared for a patient cohort, with two SPECT/CTs acquired 24 and 48 h after [^225^Ac]Ac-PSMA-I&T therapy, to evaluate their impact on kidney and lesion dosimetry.

**Results:**

In the phantom study, TDSC outperformed EWSC/no SC across all energy windows in terms of CNR, and in terms of RC for 218 and 78 keV energy windows under clinically relevant conditions. Application of PVC techniques resulted in a clear increase in RC and CNR across all energy windows. In the patient study, RBE-weighted kidney absorbed doses increased on average across all kidneys by 9 ± 4%, 30 ± 29% and 35 ± 29% for 440, 218 and 78 keV energy windows, respectively, when TDSC was applied. For lesion dosimetry, TDSC resulted in an average increase across all lesions by 16 ± 8% (218 keV) and 31 ± 30% (78 keV), and a decrease by 4 ± 8% (440 keV). In the patient study, IY-based PVC increased kidney absorbed doses by 172 ± 54%, 157 ± 45% and 146 ± 47%, for 440, 218 and 78 keV energy windows, respectively. RL-based PVC increased lesion absorbed doses by 34 ± 6%, 29 ± 8%, and 23 ± 10%, for 440, 218 and 78 keV energy windows, respectively.

**Conclusion:**

The phantom and patient studies demonstrated TDSC superiority over EWSC/no SC. PVC techniques substantially increased kidney (IY) and lesion (RL) absorbed doses, highlighting their value for post-reconstruction enhancement of ^225^Ac SPECT images.

**Supplementary Information:**

The online version contains supplementary material available at 10.1186/s40658-025-00800-0.

## Background

[^225^Ac]Ac-Prostate-Specific Membrane Antigen (PSMA) radioligand therapy (RLT) is a promising option for patients with metastatic castration-resistant prostate cancer (mCRPC), who have reached the end stages of the disease and have exhausted alternative treatment options [1–5]. Similarly, promising results have been observed with [^225^Ac]Ac-DOTA-octreotate (DOTATATE) treatment for gastroenteropancreatic neuroendocrine tumors (GEP-NETs) and other NETs [6, 7]. Quantitative single photon emission computed tomography (SPECT) imaging can assist in PSMA-targeting RLT or Peptide-Receptor-Radionuclide-Therapy, particularly for patient-specific dosimetry, post-therapeutic quality assurance and monitoring of disease progression [8]. Patient-specific dosimetry provides a means of minimizing the risk of long-term radiation toxicity, but also supports the implementation of personalised treatment schedules, i.e. adjustments of the administered activity per cycle or the number of overall therapy cycles, with the aim to optimise therapeutic efficiency [9–11].

Nevertheless, the low therapeutic activities (typically around 8 MBq) used in ^225^Ac-PSMA therapy complicate post-therapeutic SPECT imaging. ^225^Ac has two imageable daughter nuclides: ^221^Fr (218 keV) and ^213^Bi (440 keV) with gamma emission probabilities of 11.4% and 25.9%, respectively [12–14]. Additionally, several X-ray emissions in the range of 70–90 keV, primarily originating from daughter nuclides in the decay chain of ^225^Ac, along with a contribution from characteristic lead X-rays from the collimator, are detected and contribute to the SPECT image formation [15–17]. Despite the growing demand for ^225^Ac in targeted alpha therapy (TAT), SPECT imaging of ^225^Ac remains in its early stages of development [18, 19]. In addition to our previous study about SPECT imaging of mCRPC patients undergoing [^225^Ac]Ac-PSMA-I&T therapy, a recent phantom study by Tulik et al. demonstrated feasibility of quantitative SPECT imaging for glioblastomas treated with [^225^Ac]Ac-DOTA-substance P [20, 21]. Polson et al. presented a computationally efficient algorithm for high energy collimator-detector response (CDR) modelling and showed its effectiveness for ^225^Ac on both phantom and patient SPECT images [22].

The objective of this study was to investigate the impact of scatter correction and partial volume correction (PVC) techniques on post-therapeutic imaging of the three imageable energy windows of ^225^Ac (X-ray window, 218 keV and 440 keV). Energy-window (EWSC) and transmission-dependent scatter correction (TDSC) methods were investigated and compared for the 440 and 218 keV photopeaks, while for the X-ray energy window, TDSC was evaluated against reconstructed images without scatter correction (no SC). Although EWSC is well-established for SPECT imaging, it may underperform in terms of contrast-to-noise ratio (CNR) when compared to model-based techniques [23]. This difference may be more pronounced in low-count imaging scenarios, as scatter windows are typically subject to even higher noise levels compared to photopeak windows, which could lead to an overestimation of the scatter contribution [24]. This effect may be in particular of importance for the photopeak at 218 keV, showing an approximately two-fold lower emission probability compared to the photopeak at 440 keV. Hence, scatter correction in the extreme low-count regime may benefit from model-based approaches, such as TDSC, which provide noise-free scatter projections that may help reduce overall image noise and thus, e.g., improve CNR.

The partial volume effect (PVE) in SPECT imaging is primarily driven by the system’s limited spatial resolution, leading to an underestimation of activity in regions smaller than two to three times the spatial resolution [25, 26]. PVE is further enhanced in ^225^Ac SPECT imaging due to the low count statistics, which requires strong regularization to adequately suppress noise. Finally, the use of conventional high-energy (HE) collimators in ^225^Ac SPECT imaging does not fully prevent the septal penetration of the 440 keV photons, which further exacerbates the PVE. These challenges highlight the importance of PVC techniques to improve quantification accuracy and lesion detectability.

In this study, two PVC techniques were investigated for images reconstructed using TDSC. First, the Richardson-Lucy (RL) algorithm, already established for application in positron emission computed (PET) tomography, was studied [27–29]. This is an iterative, deconvolution-based PVC algorithm that does not require any prior anatomical information and is generally applied to the whole image. The algorithm was primarily developed to perform deblurring of astronomical images using a known point spread function (PSF) of the imaging system. Over time, the application of RL deconvolution found its use in PET imaging, where image restoration may be beneficial due to the inherent limitations in spatial resolution of the imaging systems. Therefore, this algorithm may be well-suited to also improve lesion quantification in SPECT imaging. In comparison, the Iterative Yang (IY) algorithm iteratively refines the activity estimates across the entire image based on prior knowledge of a regional segmentation and the system’s PSF [30]. Thus, IY-based PVC is particularly suitable for organs whose anatomical segmentations can be obtained from the computed tomography (CT) images.

In this study, EWSC and TDSC as well as both approaches for PVC were applied to phantom and patient data.

## Materials and methods

### Phantom study

#### SPECT acquisition and reconstruction

**Fig. 1:**
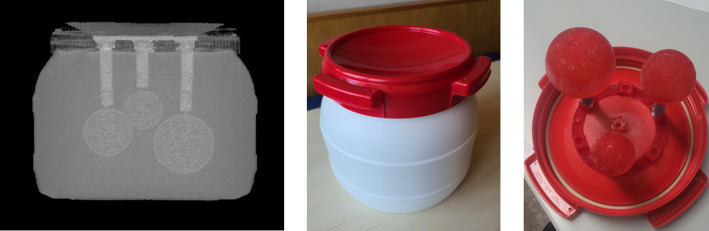
CT (left), photographs of the self-designed phantom (center) and the three fillable spheres (right)

A self-made phantom was designed for this study (Fig. 1). Three spheres with volumes of 191, 100 and 48 ml were 3D-printed and placed in a cylindrically shaped object with a total volume of 8.7 L (diameter of 25 cm). The two large spheres represent the range of typical whole kidney or cortex volumes while the smallest sphere represents large lesions. The activity concentrations in the spheres and the background were 4.2 and 0.5 kBq/ml, respectively, resulting in a foreground-to-background ratio of 8.1:1.

The phantom was measured on a Siemens Symbia T2 SPECT/CT (Siemens Medical Solutions, Erlangen, Germany) system mounted with a HE collimator. 32 projections were acquired with a matrix size of 128 × 128 pixels (4.80 × 4.80 mm^2^). The acquisition time per projection was chosen to obtain a count statistics that is expected for imaging of the kidneys around 48 h post injection (p.i.) of 8 MBq of [^225^Ac]Ac-PSMA-I&T. In the patient cohort from our previous study [20], the average kidney activity concentration was found to be 180 Bq/ml at 48 h, corresponding to a 23-fold lower activity concentration compared to the sphere activity concentration used in this study. The acquisition time per projection was therefore reduced from 210 s, which corresponds to our normal acquisition time for clinical patient imaging, to 10 s per projection. The acquisition time of 10 s per projection will be referred to as low-count (LC) case. An additional measurement was performed with 120 s per projection, which will be referred to as high-count (HC) case. Energy windows were set at 440 keV (width of 20%) for ^213^Bi, 218 keV (width of 20%) for ^221^Fr, and 78 keV (width: 50%) to cover the X-ray emissions [12, 20]. To perform EWSC, the following scatter windows were acquired: a lower adjacent scatter window of 10% width for 440 keV (dual-energy window (DEW) scatter correction); a lower and an upper scatter window at 178 keV and 267 keV (width of 20% each) for the peak at 218 keV (triple-energy window (TEW) scatter correction). Additionally, a CT scan (110 keV, slice thickness 1.25 mm) was acquired along with each of the SPECT scans.

SPECT images were reconstructed for all energy windows using an in-house maximum-a-posteriori maximum-likelihood-expectation-maximization (MAP-MLEM) algorithm using 100 iterations (penalty β = 0.01) [31]. Attenuation correction was based on the low-dose CT. Resolution modelling was based on a pre-simulated 2D PSF model [12]. After reconstruction, all SPECT images were filtered for noise suppression using a Gaussian filter with a 30 mm full-width at half maximum (FWHM), consistent with prior studies [12, 20].

The calibration factors to convert measured counts per second per voxel (cps/voxel) to activity concentration (Bq/ml) were determined for each energy window and scatter correction method separately. Calibration measurement was conducted using the same phantom but with the 3D-printed spheres removed to ensure a homogeneous activity distribution. The background activity concentration was 0.5 kBq/ml, identical to that used in the phantom study. Imaging was conducted using the HC protocol (total of 32 projections, 120 s per projection). A spherical volume of interest (VOI) with a diameter of 16 cm was placed centrally within the phantom to determine the mean cps/voxel for calibration. Reconstruction followed the same protocol as described above, including the post-reconstruction 30 mm FWHM Gaussian filtering.

#### Scatter correction

The performance of EWSC and TDSC for the 440 and 218 keV photopeaks was assessed by analyzing the resulting recovery coefficients (RC) and CNR. For the X-ray (78 keV) energy window, TDSC was compared with no SC rather than EWSC, as the use of EWSC might not be suitable for this energy window. Analysis of the simulated energy spectrum for primary and scattered photons indicates that triple-energy-window based scatter correction, i.e. approximating the number of scattered counts by a trapezoid, based on adjacent scatter windows is not straight forward (Figure A1 in supplementary information). Additionally, the 78 keV energy window consists of multiple overlapping X-ray emissions from multiple daughters and characteristic lead X-rays from the collimator, rather than a single distinct emission peak. A similar issue was observed by Elschot et al. in their study of ^166^Ho SPECT imaging, where the major photopeak at 81 keV, closely matching the 78 keV energy window of ^225^Ac, was affected by a characteristic lead X-ray peak at 74 keV, impeding the effectiveness of EWSC [32].

According to the method proposed by Sohlberg et al., the core principle of TDSC within quantitative reconstruction is to convolve scatter kernels with the image estimate after iteration k to generate a scatter image, which can then be forward projected to create scatter projections for iteration k + 1 [33]. To generate the scatter kernels required for TDSC, the SIMIND Monte Carlo program (ver. 6.2) was used to simulate line profiles behind water slabs of varying thicknesses (2–40 cm in steps of 2 cm) for all three energy windows [34]. Validation measurements for simulated line profiles were performed for selected water thicknesses and are provided in the supplementary information (Figure A2). For all slab thicknesses, the tails of the scatter signal were fitted using a mono-exponential function. Given the negligible change in slope of the mono-exponential functions above depths of 40 cm (changes in slopes are less than 3% for every additional 2 cm of slab thickness), in instances where the depth of the patient exceeded 40 cm, the mono-exponential function corresponding to a depth of 40 cm was used. Scatter-to-primary fractions ($\:\text{S}{\text{F}}_{\text{S}/\text{P}}$) were obtained from SIMIND and fitted as a function of attenuation path length using the following model:

1 $$\:\text{S}{\text{F}}_{\text{S}/\text{P}}=\text{A}-\text{B}\:{\left[{\text{e}}^{-\sum\:_{\text{i}=\text{j}}^{\text{s}\text{k}\text{i}\text{n}}{{\upmu\:}}_{i}\varDelta\:}\right]}^{{\upgamma\:}}-1,$$

where A, B, and γ are the coefficients obtained from the fit, $\:{{\upmu\:}}_{\text{i}}$ is the linear attenuation coefficient for the voxel i and $\:\varDelta\:$ corresponds to the voxel size. The summation is performed over all voxels along a straight line (or ray) from the voxel of interest j, located at some depth within patient, toward the patient surface (skin) in the direction of the detector [33].

The fitted mono-exponential functions were used to generate depth-dependent scatter kernels. To generate the scatter image for iteration k + 1, each slice of the current image estimate was convolved with the scatter kernel, corresponding to the depth from the current slice to the surface of the patient. In the next step, the attenuation path length of each voxel of the current slice was used to determine the corresponding scatter-to-primary fraction, which was then applied as a scaling factor for that voxel. These steps were performed independently for each projection angle.

#### Partial volume correction

The RL and IY algorithms were implemented in Python 3.11 using the Scikit-image package (version 0.24.0) and a PETPVC toolbox of the Nipype (Neuroimaging in Python Pipelines and Interfaces) library (version 1.8.4), respectively [35, 36]. Both RL and IY algorithms were applied as post-processing to the reconstructed SPECT images (with TDSC). To determine the PSF of the imaging system, it was assumed that the reconstructed images suffer from a spatially invariant and isotropic 3D Gaussian blur. The standard deviation for the 3D Gaussian function (and thus that of the PSF) was determined for each energy window by applying Gaussian filters with varying standard deviations (ranging from 0.5 to 2.0 cm in steps of 0.25 cm) to the 3D ground truth image of the 3D-printed phantom. The 3D ground truth image was obtained from the CT of the 3D-printed phantom, in which the three spheres and the phantom itself were manually segmented. The segmented regions were then filled with activity concentrations as used in the measurements (4.2 and 0.5 kBq/ml in the spheres and background, respectively), resulting in a 3D ground truth image. A matched-filter analysis was then conducted, with spherical VOIs defined around each of the phantom’s spheres [37]. The radii of these VOIs were increased by 2 cm beyond the radii of the original spheres. This was done to cover for the spread due to the Gaussian blur, whilst also avoiding overlapping with other spheres. The root mean squared error (RMSE) for each VOI was computed by comparing the Gaussian filtered ground truth image and the reconstructed phantom image from the HC measurement with 32 projections (without Gaussian post-filter). The standard deviation that minimised the summed RMSE across all spheres was selected.

Afterwards, both IY and RL algorithms were evaluated for PVC on the LC SPECT of the phantom with 32 projections, which is equivalent to our current patient imaging protocol. Since IY-based PVC requires knowledge on the region segmentations, the CT-based segmentations of the phantom’s spheres were used. For RL-based PVC, the optimum number of iterations that would result in the best balance between PVC (yielding highest RC in VOIs) and added noise (CNR) was determined. For IY-based PVC, the default of 10 iterations, as defined in the PETPVC toolbox, was used. For both IY and RL, it was assessed whether Gaussian filtering should be applied before or after PVC. This was based on the achieved RC, CNR and preservation of the VOI shapes. If applied before PVC, the PSF for PVC was calculated using quadrature summation of the standard deviations of the imaging system’s PSF and Gaussian filter. All optimisations were performed separately for each energy window. For both IY and RL algorithms, RC and CNR for all spheres were calculated using CT-based segmentations of the phantom’s spheres. Additionally, for the RL-based PVC, RC and CNR for the smallest sphere were also obtained using isocontour segmentations, as this is a common way for lesion segmentation in routine dosimetry. The methodology for generating isocontour segmentations is explained in Sect. 2.2.

#### Image analysis metrics

**Fig. 2:**
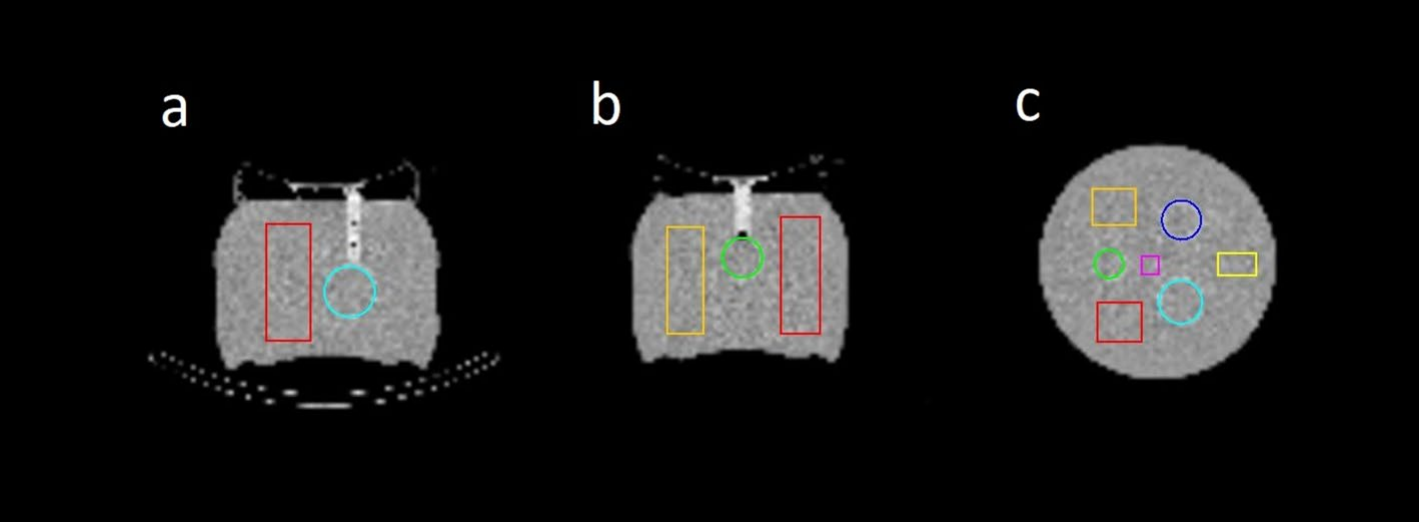
CT scan of the phantom with segmented 3D-printed spheres (blue: 191 ml, cyan: 100 ml, green: 48 ml) and rectangular background VOIs used for CNR calculation (magenta, yellow, orange, red) in (**a**) axial, (**b**) sagittal, and (**c**) coronal views. The volumes of the background VOIs are 54 ml, 145 ml, 260 ml and 280 ml for magenta, yellow, orange and red, respectively

The phantom CNR and RC were analyzed using PMOD (Version 3.609, PMOD Technologies, Zurich, Switzerland). The CNR was calculated according to the following formula (Eq. 2):

2 $$\:C\text{N}\text{R}=\frac{{{\upmu\:}}_{\text{V}\text{O}\text{I}}-{{\upmu\:}}_{\text{B}\text{a}\text{c}\text{k}\text{g}\text{r}\text{o}\text{u}\text{n}\text{d}}}{{{\upsigma\:}}_{\text{B}\text{a}\text{c}\text{k}\text{g}\text{r}\text{o}\text{u}\text{n}\text{d}}},$$

where $\:{{\upmu\:}}_{\text{V}\text{O}\text{I}}$ is the mean activity concentration in the spheres and $\:{{\upmu\:}}_{\text{B}\text{a}\text{c}\text{k}\text{g}\text{r}\text{o}\text{u}\text{n}\text{d}}$ and $\:{{\upsigma\:}}_{\text{B}\text{a}\text{c}\text{k}\text{g}\text{r}\text{o}\text{u}\text{n}\text{d}}$ are the mean activity concentration and the corresponding standard deviation in the background. CT-based sphere segmentations (with an additional isocontour segmentation applied to the smallest sphere) were used to obtain $\:{{\upmu\:}}_{\text{V}\text{O}\text{I}}$. For $\:{{\upmu\:}}_{\text{B}\text{a}\text{c}\text{k}\text{g}\text{r}\text{o}\text{u}\text{n}\text{d}}$ and $\:{{\upsigma\:}}_{\text{B}\text{a}\text{c}\text{k}\text{g}\text{r}\text{o}\text{u}\text{n}\text{d}}$, the average of the means and the standard deviations from the four VOIs were calculated, with the VOIs evenly distributed across the background compartment at sufficient distances from the spheres to avoid signal contamination (Fig. 2).

The sphere RC (without spill-over correction) was calculated using Eq. 3:

3 $$\:\text{R}\text{C}=\frac{{\text{A}\text{C}}_{\text{V}\text{O}\text{I}}}{{\text{A}\text{C}}_{\text{T}\text{r}\text{u}\text{e}}}\times\:100\%,$$

where $\:{\text{A}\text{C}}_{\text{V}\text{O}\text{I}}$ and $\:{\text{A}\text{C}}_{\text{T}\text{r}\text{u}\text{e}}$ are the measured and the true activity concentrations in the spherical VOIs, respectively.

### Patient study

#### SPECT data acquisition and reconstruction

The optimal post-processing workflow, as determined by the phantom study, was subsequently evaluated in a retrospective patient cohort consisting of 5 patients diagnosed with mCRPC and treated with 7.7 ± 0.2 MBq [^225^Ac]Ac-PSMA-I&T (ethics approval 22–0544). All patients gave written consent to undergo RLT. All data has been irreversibly anonymized before evaluation. The patient data included two post-therapeutic abdominal SPECT/CTs acquired at 24 and 48 h p.i. SPECT raw data were acquired with a total of 32 projections and an acquisition time of 210 s per projection. Additionally, a low-dose CT scan (110 keV, CareDose, slice thickness 3 mm) was performed after each SPECT scan. All other acquisition and reconstruction parameters were consistent with those for the phantom study (Sect. 1.1).

#### Impact of scatter and partial volume corrections on dosimetry

Scatter correction was evaluated by comparing TDSC and EWSC/no SC (without PVC) in the patient cohort. The optimised RL and IY PVC algorithms were applied to the reconstructed SPECT images (with TDSC) to study the impact of PVC on lesion (RL) and kidney (IY) dosimetry. Image analysis was performed using PMOD (Version 3.609, PMOD Technologies, Zurich, Switzerland). For simplicity and as the focus of this study was the impact of scatter correction methodology and PVC on quantification, local energy deposition of all ^225^Ac daughters was assumed and dosimetry was evaluated using each energy window separately as a surrogate for the overall energy depositions. This means that the spatial distribution of ²²⁵Ac and all subsequent daughters is described by SPECT imaging of either the ²¹³Bi, ²²¹Fr, or X-ray energy windows. The corresponding S-value was taken from the open-access online resource OpenDose and includes the entire decay chain [38]. Kidney and lesion RBE-weighted absorbed doses (RBE: relative biological effectiveness) were estimated based on the MIRD formalism using a RBE of 5 [39, 40]. For each patient, the kidneys and all lesions visible in the abdominal region were evaluated (no lesions were available in the field-of-view for patient 1). The lesions were segmented using an isocontour of the maximum tissue intensity. The exact percentage for the isocontour was determined by identifying the isocontour that resulted in the best agreement with the true volume for the smallest sphere in the phantom study, as the volume of the smallest sphere is closest to the upper range of typical lesion volumes visible in ^225^Ac-SPECT imaging [20]. The kidneys were segmented on the CT scan acquired 24 h p.i. Both, the segmentations for lesions and kidneys, were transferred to the SPECT acquired at 48 h p.i. and manually adjusted in case of misalignment. Since only two post-therapeutic images were available, a mono-exponential function was used to model the time-activity-curves. Additionally, the RBE-weighted absorbed doses for the kidneys and lesions were statistically compared between the two scatter correction methods using Wilcoxon signed-rank test (Python 3.11, Scipy package ver. 1.16.0 [41])

## Results

### Phantom study

#### Scatter correction

**Fig. 3:**
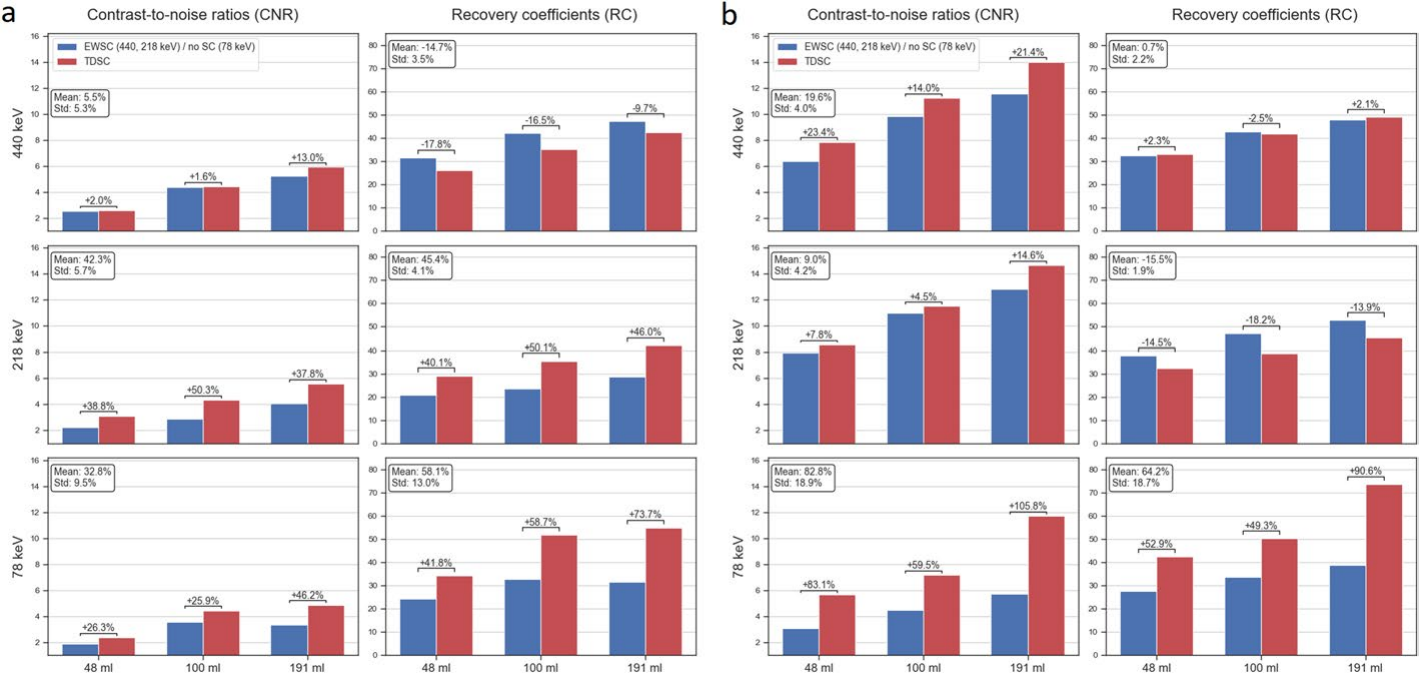
CNR and RC comparison of TDSC and EWSC (440, 218 keV) / no SC (78 keV) for all three energy windows for the LC (**a**) and HC (**b**) cases. The figure includes the percentage changes for CNR and RC for each of the spheres and peaks from EWSC/no SC to TDSC. Additionally, in the text boxes, the mean and standard deviations (Std) of the percentage changes for CNR and RC across all spheres for each of the peaks are shown. All images were filtered using 30 mm FWHM Gaussian filter. No PVC was applied

In Fig. 3, CNR and RC for TDSC and EWSC/no SC are presented for the three energy windows and for the LC (a) and HC (b) cases with 32 projections. No PVC was applied to the SPECT images. TDSC improved CNR across all energy windows and spheres for both HC and LC cases. Regarding RC, TDSC showed substantial improvements in the X-ray energy window for both LC and HC cases. For the 218 keV energy window, TDSC resulted in substantially higher RC in the LC case, whereas yielded lower RC than EWSC in the HC case. For the 440 keV energy window, RC were higher using the EWSC in the LC case and comparable between methods in the HC case.

#### Partial volume correction

In Table 1, the optimal parameters for the investigated PVC methods are presented. Gaussian filtering was carried out after RL-based PVC and before IY-based PVC.

**Fig. 4:**
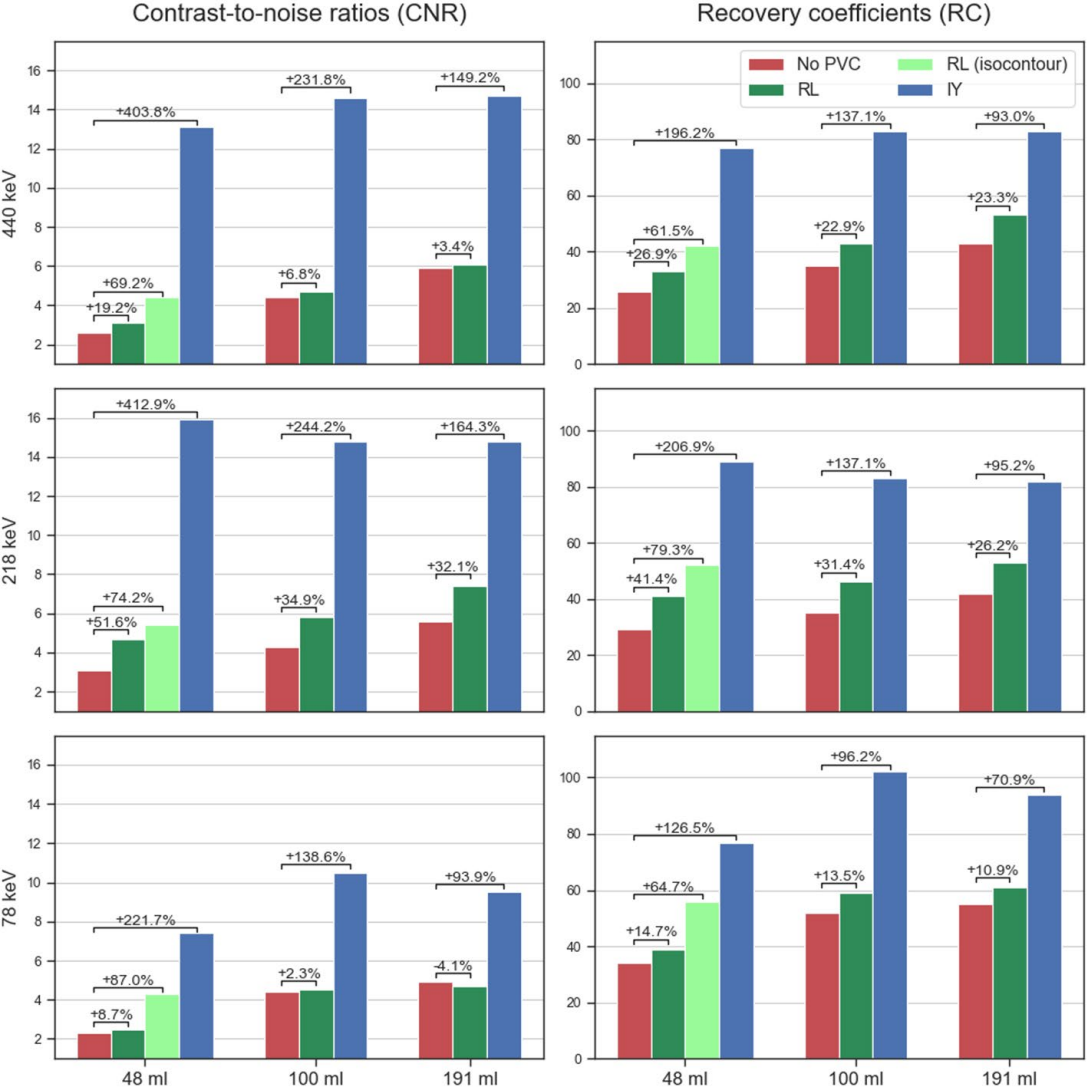
RC and CNR for no PVC, RL and IY for all spheres in the phantom. For the smallest sphere, RC and CNR obtained using isocontour segmentations for RL are also shown. The figure includes the percentage changes for CNR and RC for each of the spheres and peaks between no PVC and the PVC methods

**Fig. 5:**
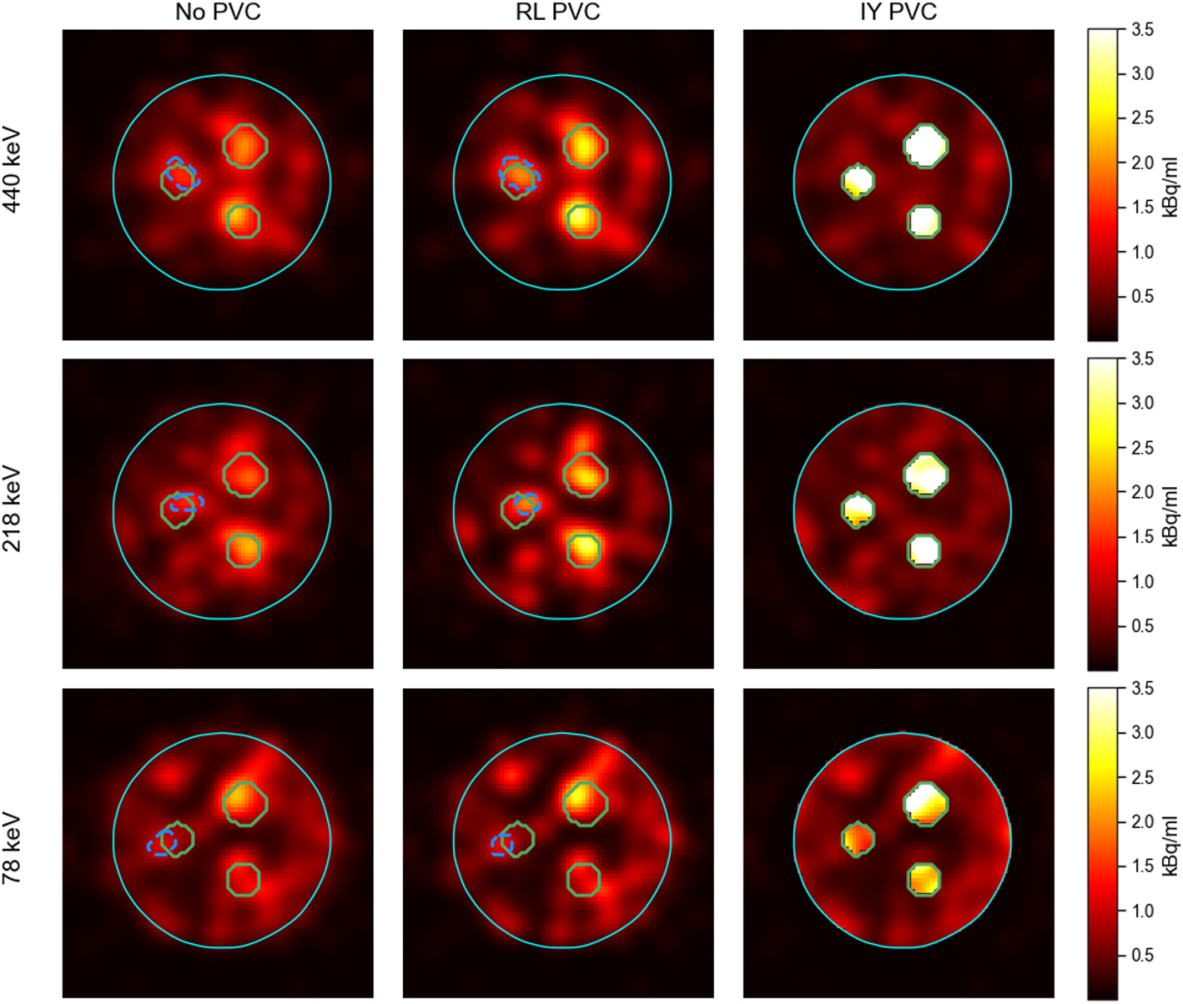
Slices from post-filtered SPECT images of the phantom using no PVC, RL and IY (Gaussian post-filter, 30 mm FWHM) for the three energy windows. The locations of the phantom spheres are shown with green solid lines, the isocontour segmentations for the smallest sphere for RL-based and non-PVC images with dashed blue lines, and the phantom’s background volume with cyan solid line

In Fig. 4, RC and CNR for all spheres in the phantom are presented for no PVC, RL and IY PVC images (LC, 32 projections), obtained using the parameters from Table 1. Data used for Fig. 4 are provided in the supplementary information (Table A1). In Fig. 5, slices of the corresponding phantom SPECT are shown. For RL-based PVC-corrected SPECT images, isocontours of 65% were used for the 440 keV and 218 keV energy windows, while a threshold of 60% was applied to the 78 keV window. For non-PVC-corrected SPECT images, an 80% isocontour threshold was used for all three energy windows. The same isocontour thresholds were also applied for lesion segmentation in the patient study. The mean percentage increase in RC across all spheres for RL and IY were 24 ± 2% and 142 ± 52% for 440 keV, 33 ± 8% and 146 ± 56% for 218 keV, and 13 ± 2% and 98 ± 28% for 78 keV, respectively. When using isocontour-based segmentation for the smallest sphere in both no PVC and RL images, RL resulted in the percentage increase in RC of 27%, 37% and 10% for 440, 218 and 78 keV energy windows, respectively. The mean percentage increases in CNR across all spheres for RL and IY were 10 ± 8% and 262 ± 130% for 440 keV, 40 ± 11% and 274 ± 127% for 218 keV, 2 ± 6% and 151 ± 65% for 78 keV, respectively. When isocontour segmentation was applied to the smallest sphere in both no PVC and RL images, RL yielded a CNR increase of 42%, 59% and 16% for 440, 218 and 78 keV energy windows, respectively.

Both IY and RL PVC resulted in a noticeable increase of RC and CNR across all energy windows. Overall, IY-based PVC resulted in a greater improvement in RC and CNR compared to RL-based PVC. This can be attributed to the fact that the IY algorithm uses segmentations of the VOIs and the phantom, providing it with prior knowledge of where the activity is expected. For the smallest sphere, the quantitative enhancement was more pronounced with isocontour-based segmentation than with CT-based segmentation, as the isocontour approach at least partially compensates for distortions in the activity distribution.

**Table 1:** Optimized parameters for RL and IY PVC. Gaussian filtering (FWHM of 30 mm) was performed after RL-based PVC and prior to IY-based PVC

Energy	Richardson-Lucy (RL)		Iterative Yang (IY)	
	PSF σ [cm]	No. of iterations	PSF σ [cm]	Combined PSF σ including σ = 1.27 cm for noise suppression prior to IY [cm]
440 keV	1.50	7	1.50	1.96
218 keV	1.50	15	1.50	1.96
78 keV	1.00	10	1.00	1.62

### Patient study

RBE-weighted kidney and lesion absorbed doses for all three energy windows, reconstructed with TDSC and EWSC/no SC, along with Wilcoxon signed-rank *p*-values, are shown in Table 2. For the RBE-weighted kidney absorbed doses, TDSC resulted in statistically significant mean per-kidney increase of 9 ± 4%, 30 ± 29%, and 35 ± 29% for the 440, 218 and 78 keV energy windows, respectively. For the RBE-weighted lesion absorbed doses, TDSC yielded statistically significant mean per-lesion increase of 16 ± 8% for 218 keV and 31 ± 30% for 78 keV energy windows, but a decrease of 4 ± 8% for 440 keV photopeak (*p*-value = 0.074). Some examples of the reconstructed patient images using EWSC/no SC and TDSC are shown in supplementary information (Figure A3).

**Fig. 6:**
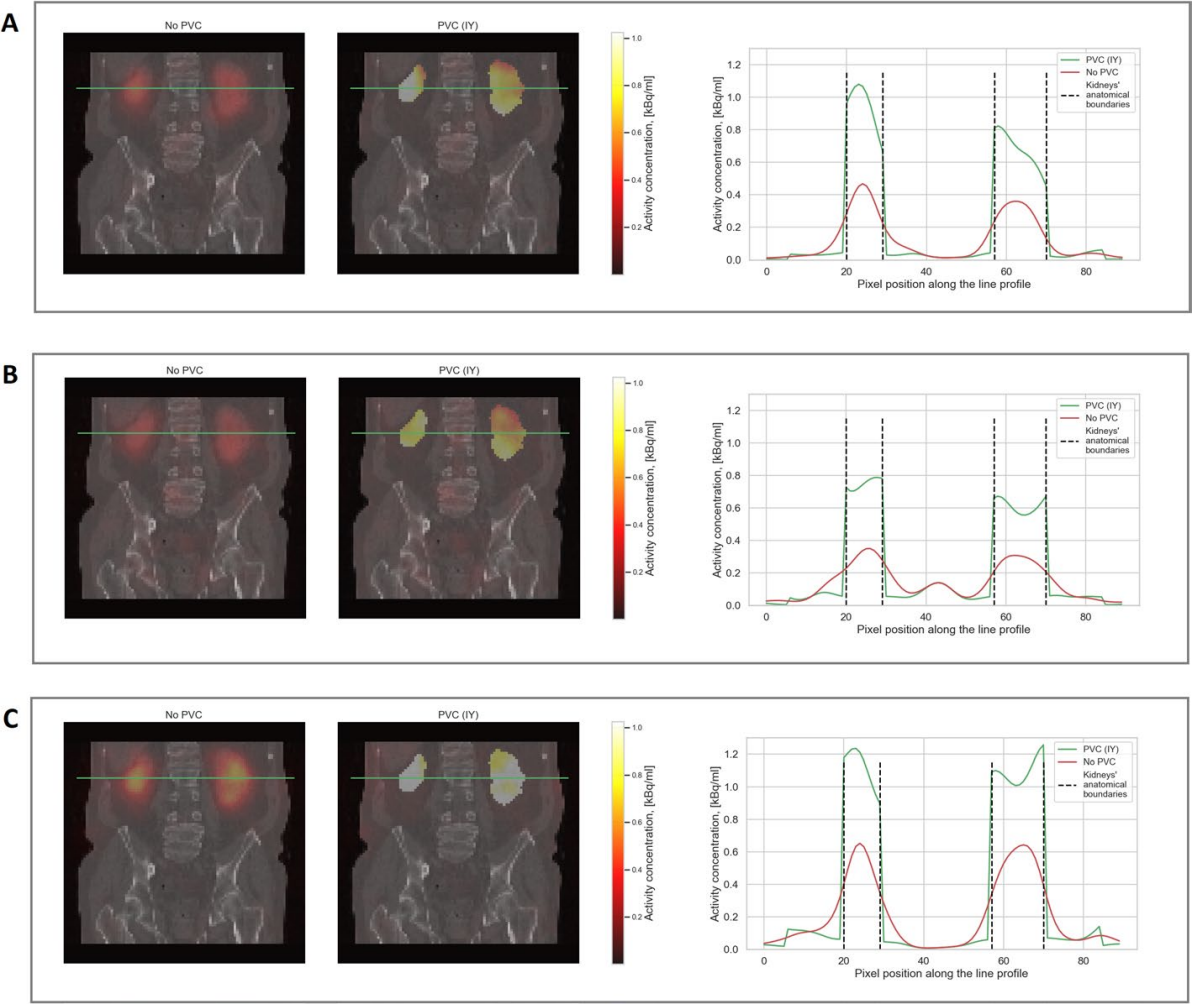
SPECT/CT slices from all three energy windows (**A**: 440 keV, **B**: 218 keV, C: X-rays) of patient 1 (Fig. 8) using no PVC and IY. The green lines on the SPECT/CT images indicate the location through which the activity profiles were taken. All SPECT images were filtered using 30 mm FWHM Gaussian filter

**Fig. 7:**
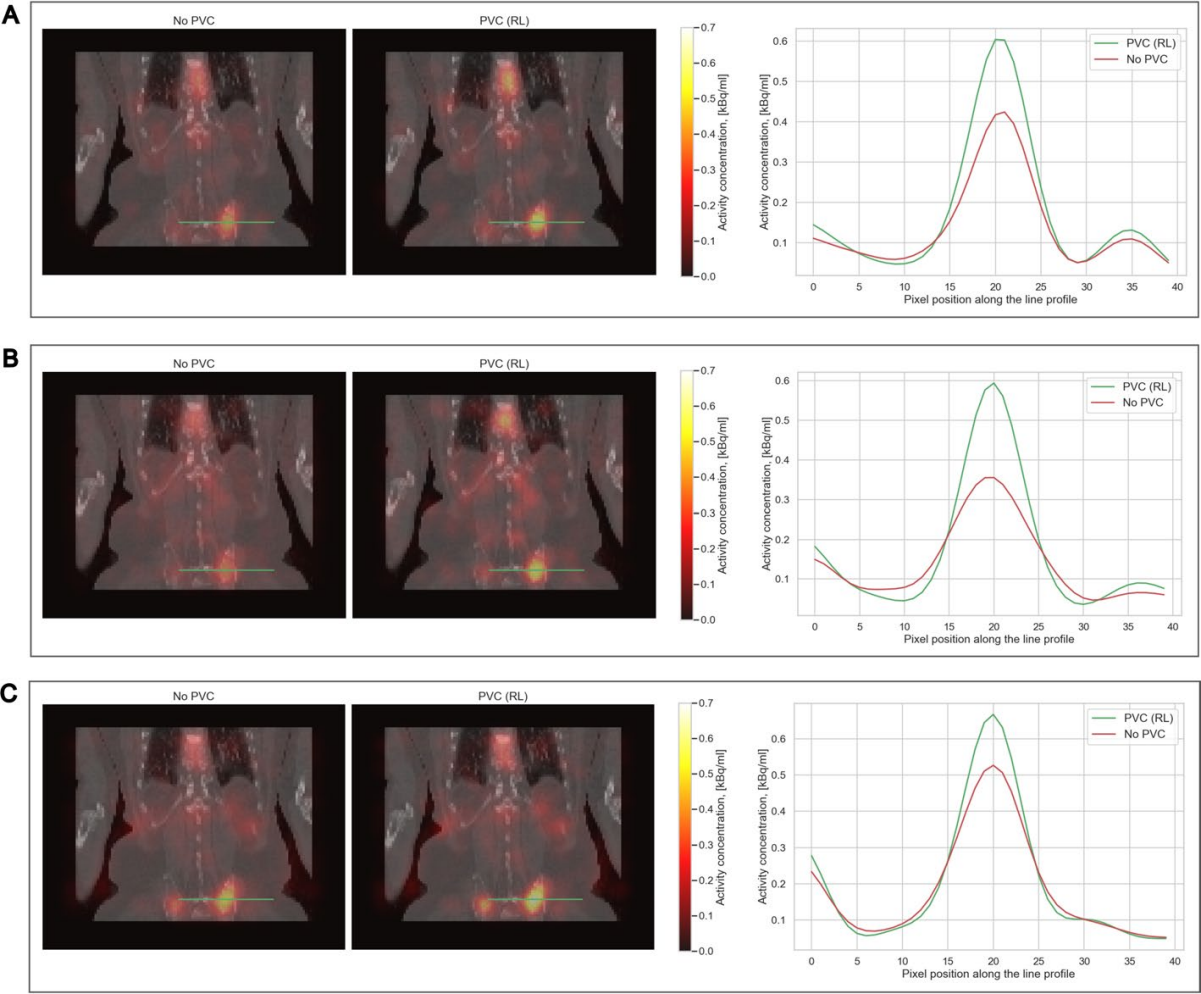
SPECT/CT slices from all three energy windows (**A**: 440 keV, **B**: 218 keV, C: X-rays) patient 5 (Fig. 8) using no PVC and RL. The green lines on the SPECT/CT images indicate the location through which the activity profiles were taken. All SPECT images were filtered using 30 mm FWHM Gaussian filter

**Fig. 8:**
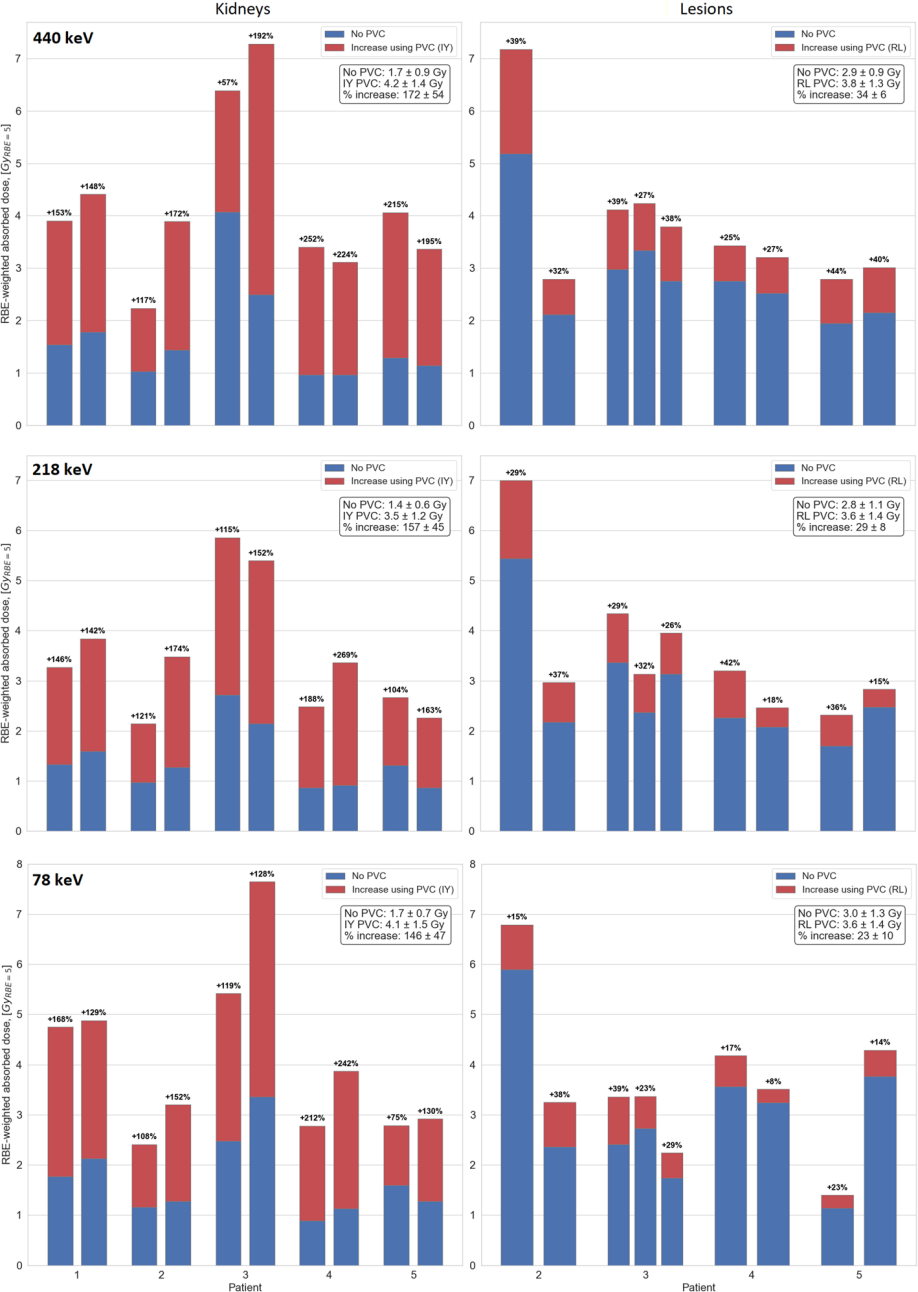
RBE-weighted kidney (left) and lesion (right) absorbed doses for all three energy windows with and without PVC. The increase of RBE-weighted absorbed doses, obtained when using PVC SPECT, is shown as red bars and the resulting RBE-weighted absorbed doses are represented by the sum of the blue and red bar heights

Figures 6 and 7 visualise some exemplary patient data comparing IY-based PVC for the kidneys and RL-based PVC for the lesions to SPECT without PVC. Figure 8 presents the RBE-weighted kidney and lesion absorbed doses with and without PVC applied. In this figure, the blue bars represent the absorbed doses without PVC, while the red bars show the increase in absorbed dose attributable to PVC. The total height of each combined blue and red bar corresponds to the absorbed dose after applying PVC. Further information on the kidney and lesion dosimetry with and without PVC is provided in the supplementary information (Tables A2-A4), including results for kidney dosimetry obtained for RL-based PVC for comparison. The use of RL- and IY-based PVC techniques led to a clear increase in RBE-weighted absorbed doses for lesions and kidneys, respectively, across all energy windows. In addition, the reduction of PVE achieved through PVC techniques resulted in enhanced detectability of kidneys and lesions in patient images.

**Table 2:** Mean kidney and lesion RBE-weighted absorbed doses for the patient cohort, calculated using TDSC and EWSC (440, 218 keV) / no SC (78 keV), are presented for all three energy windows. No PVC was applied

Energy	RBE-weighted kidney absorbed dose (mean ± std), [Gy]		*p*-value	RBE-weighted lesion absorbed dose (mean ± std), [Gy]		*p*-value
	TDSC	EWSC/no SC		TDSC	EWSC/no SC	
440 keV	1.7 ± 0.9	1.5 ± 0.7	0.0020	2.9 ± 0.9	3.0 ± 1.1	0.0740
218 keV	1.4 ± 0.6	1.1 ± 0.7	0.0059	2.8 ± 1.1	2.4 ± 0.8	0.0039
78 keV	1.7 ± 0.7	1.3 ± 0.6	0.0254	3.0 ± 1.3	2.2 ± 0.7	0.0156

## Discussion

The aim of this study was to examine the impact of scatter correction and PVC techniques on post-therapeutic SPECT imaging of ^225^Ac. The scatter correction techniques were investigated using a phantom study, primarily focusing on RC and CNR, and a patient study, focusing on kidney and lesion dosimetry. For the patient study, the dosimetry of the kidneys and lesions was also compared between SPECT images with and without PVC.

The comparison of the TDSC and EWSC methods (no SC for 78 keV) showed that TDSC consistently yielded higher CNR across all spheres and energy windows for both LC and HC cases (Fig. 3), in particular for 218 keV and 78 keV (mean increase of 42% and 33%, respectively). For RC, however, the results were more dependent on energy window and acquisition time. RC were substantially higher using TDSC for the X-ray energy window for both HC and LC cases (mean increases of 64% and 58%, respectively). For the 218 keV energy window, TDSC yielded considerably higher RC in the LC case (mean increase of 45%), while in the HC case, RC were higher using EWSC (mean increase of 15%). This may reflect a count-level dependence of the calibration factor when using EWSC for 218 keV energy window, resulting in notably higher RC in the HC compared to the LC case. For the 440 keV peak, EWSC produced higher RCs in the LC case (mean increase of 15%) and comparable results in the HC case (mean difference within 1%). In the patient study, RBE-weighted kidney and lesion absorbed doses were statistically significantly higher for TDSC than for EWSC/no SC reconstructed images for both the 218 and 78 keV energy windows. For the 440 keV energy window, RBE-weighted kidney absorbed doses were also significantly higher for TDSC, while no statistically significant difference was found for RBE-weighted lesion absorbed doses (Table 2).

The greater improvement at 218 keV energy window for the LC case is likely due to its inherently higher noise resulting from lower photon yield and significant downscatter of the 440 keV photons, making TDSC more impactful. This finding is consistent with the previous studies, which have shown that model-based scatter correction methods outperform EWSC in terms of noise properties [23]. Conversely, the 440 keV photopeak is subject to less scatter signal in the energy window and consequently requiring less scatter correction, and hence, the use of TDSC yielded comparable results to EWSC. One limitation of this study is that scatter correction for the 440 keV energy window was performed using the DEW method, despite the presence of downscatter from higher-energy photons of ^209^Tl into the photopeak window. This constraint arose from the Symbia SPECT/CT system’s limitation of allowing a maximum of six energy windows to be measured simultaneously. Nonetheless, it remains valid that scatter projections obtained using EWSC method are severely affected by noise. For the 78 keV energy window, TDSC also demonstrated significant improvement, as it effectively removes scattered contamination, thus enhancing CNR and facilitating more accurate quantification. To conclude, both the phantom LC case, which reflects clinically relevant imaging conditions, and patient studies demonstrated that TDSC significantly increased activity and absorbed dose quantifications compared to EWSC for the 218 and 78 keV energy windows, while providing comparable quantification capabilities for the 440 keV energy window. Further comparison with full MC-based scatter correction methods could be beneficial, which may outperform, for example, analytical models in highly heterogeneous and geometrically complex regions [23].

In this study, the comparison of the scatter correction and PVC methods was limited to a single phantom configuration with a fixed foreground-to-background ratio and activity level, being imaged for two count scenarios (HC and LC (clinically expected case at 48 h p.i.)). Repeated phantom imaging with varying foreground-to-background ratios and spheres focused on a range of lesion volumes would help assess the variability and robustness of the methods. Future studies could also incorporate 3D-printed organ-specific phantoms and explore a broader range of activity concentrations (e.g. reflecting typical activities in the kidneys 24 h p.i.) to improve generalisability.

For the application of PVC techniques, the stage at which Gaussian filtering was performed was determined based on visual inspection. It was determined that Gaussian filtering should be carried out after RL-based PVC to allow for more consistent VOI shape preservation and overall visual consistency with the images without PVC. For the IY-based PVC, as the target regions are predefined, the shapes are inherently maintained; thus, Gaussian filtering for noise suppression was conducted before IY. Moreover, Gaussian filtering applied after IY would degrade the sharp boundaries of the segmented regions attained by IY, thereby impairing the achieved PVC.

The use of PVC techniques has demonstrated significant improvements in terms of RC, CNR and qualitative enhancements, i.e. detectability of hot regions, in the phantom study (Figs. 4 and 5). In the patient study, the use of PVC techniques has likewise resulted in a notable increase in the RBE-weighted absorbed doses for both the kidneys and the lesions (Fig. 8). Moreover, PVC has led to improved detectability and delineation of kidneys and lesions in the reconstructed images (Figs. 6 and 7).

A primary limitation of this study is the assumption that the PSF of the imaging system is Gaussian and remains constant throughout the entire image. While this assumption simplifies the modelling process and the implementation of PVC, it may not capture the full complexity of distance-dependent blurring introduced by the imaging system. The latter is especially true for ^225^Ac SPECT imaging due to pronounced septal penetration and associated star artefacts. Although this study used a 2D resolution compensation model that accounts for distance-dependent detector blur, septal penetration and also collimator scatter, the restoration of an invariant spatial resolution and full correction of anisotropic blur may be imperfect. Additionally, the optimal PSF was determined based on a phantom with a given foreground-to-background ratio, which may not fully generalize to different uptake and geometrical scenarios. A more comprehensive analysis of the optimal PSF under diverse imaging conditions is necessary to assess the robustness of the investigated PVC methods. However, this first investigation of RL- and IY-based PVC techniques and the associated parameter optimization was conducted using a phantom scenario with clinically realistic count levels.

PVC has already been investigated for ^177^Lu SPECT imaging. Liu et al. reported an increase in lesion (*n* = 40) activity of 63.5 ± 25.2% using the iterative deconvolution-based Van-Cittert algorithm and an increase of 51.3 ± 7.4% in the kidney (*n* = 80) activities when using IY technique for ^177^Lu-SPECT imaging for 10 clinical patients [42]. Phantom studies from both this investigation and that of Liu et al. demonstrated notable improvements in activity quantification accuracy using either iterative deconvolution-based or IY-based PVC techniques, applied post-reconstruction to the SPECT images. Future research could concentrate on deep learning (DL)-based PVC techniques, which may be promising as demonstrated for a broader application in post-therapeutic ^177^Lu-SPECT imaging by Leube et al. [43].

## Conclusion

The impact of scatter correction and PVC techniques on the quantitative and qualitative quality of ^225^Ac SPECT imaging was investigated using a phantom and a patient study. For the phantom imaged using acquisition parameters reflecting a clinical protocol (LC case), TDSC resulted in higher CNR than EWSC/no SC across all three energy windows and higher RC for the 218 and 78 keV energy windows. In the patient study, TDSC yielded higher RBE-weighted kidney absorbed doses for all three energy windows. RBE-weighted lesion doses were significantly higher for the 218 and 78 keV energy windows, while they remained comparable for the 440 keV energy window. For all energy windows, the application of RL- and IY-based PVC techniques significantly enhanced the RC and CNR of the VOIs in the phantom images and resulted in a substantial increase in the RBE-weighted lesion (RL) and kidney (IY) absorbed doses in the patient study. These findings highlight that PVC techniques are a valuable tool for the post-reconstruction enhancement of ^225^Ac images.

## Supplementary Information

Below is the link to the electronic supplementary material.

Supplementary Material 1

## Data Availability

Please contact the corresponding author.

## Abbreviations

CDR: Collimator-detector response
CNR: Contrast-to-noise ratios
CT: Computed tomography
DEW: Dual-energy window scatter correction
DL: Deep learning
DOTATATE: DOTA-Octreotate
EWSC: Energy-window based scatter correction
FWHM: Full-width-at-half-maximum
GEP-NETs: Gastroenteropancreatic neuroendocrine tumors
HC: High count case
HE: High-energy collimator
HU: Hounsfield Units
IY: Iterative Yang
LC: Low count case
MAP-MLEM: Maximum-A-Posteriori Maximum-Likelihood-Expectation-Maximization
mCRPC: Metastatic castration-resistant prostate cancer
no SC: No scatter correction
PET: Positron emission tomography
PSF: Point spread function
PSMA: Prostate-specific membrane antigen
PVC: Partial volume correction
PVE: Partial volume effect
RBE: Relative biological effectiveness
RC: Recovery coefficient
RL: Richardson Lucy
RLT: Radioligand therapy
RMSE: Root mean squared error
SPECT: Single photon emission computed tomography
TAT: Targeted alpha therapy
TDSC: Transmission-dependent scatter correction
TEW: Triple-energy window scatter correction
STD: Standard deviation
VOI: Volume-of-interest
