# Supplementary material for: Comparison of scatter and partial volume correction techniques for quantitative SPECT imaging of 225Ac
Source: EJNMMI Phys. 2025 Oct 2;12:88. doi: 10.1186/s40658-025-00800-0 (PMC12491142; doi:10.1186/s40658-025-00800-0)
Supplement: Supplementary file 1 — Supplementary Material 1 [file 40658_2025_800_MOESM1_ESM.docx]

## Simulated emission spectrum of ^225^Ac





Figure A1: Simulated emission spectrum of ^225^Ac for a line source behind a 4 cm slab of water. The solid line indicates the total signal, while the dashed line shows the scattered signal. The green regions indicate the photopeak energy windows, while the red regions indicate the scattered energy windows.

## Validation measurements for SIMIND line profile simulations

##
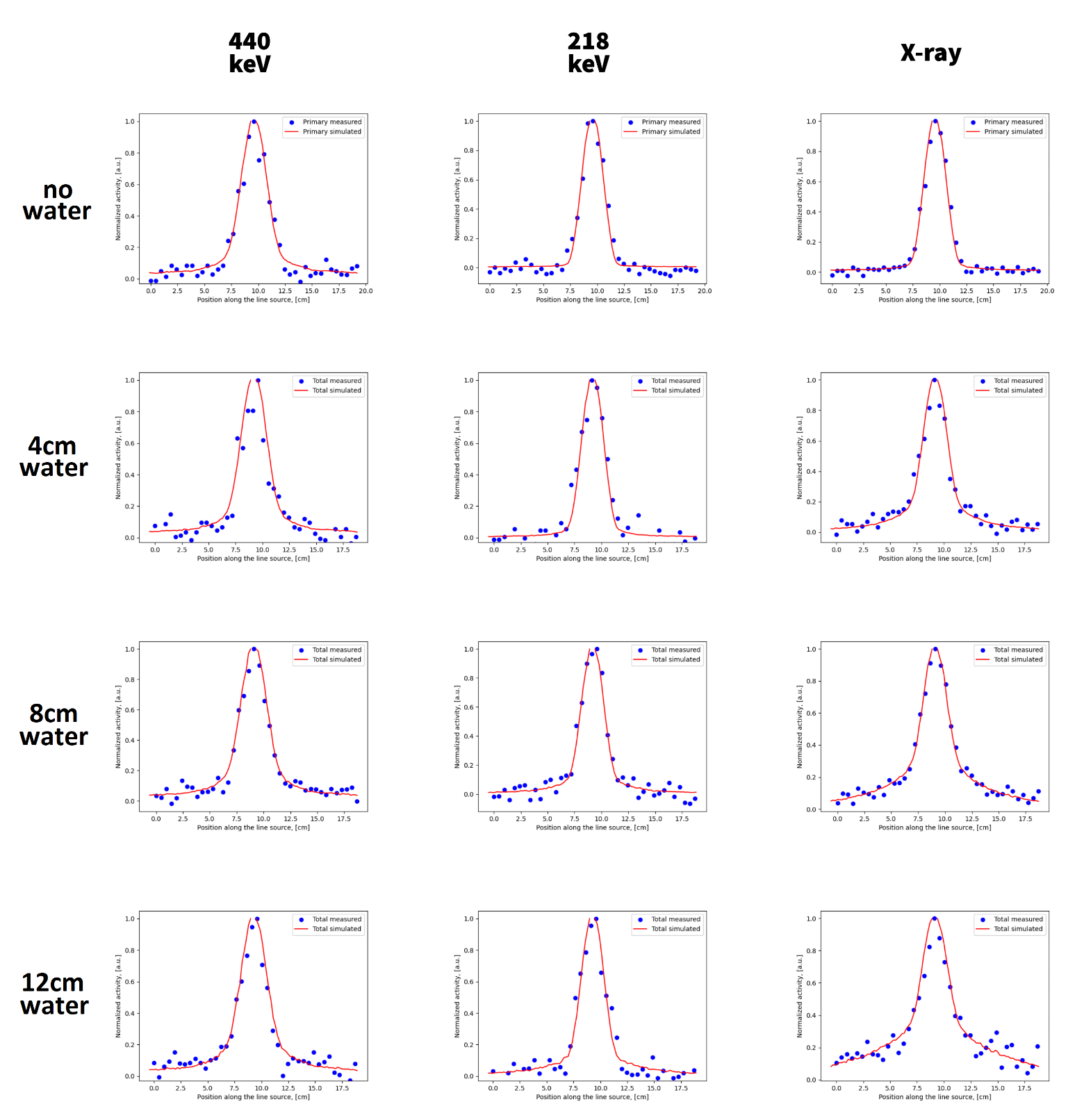


Figure A2: Planar validation measurements for SIMIND line profile simulations. A small glass tube (diameter of 1 mm and length of 6 cm) containing 20 kBq of ^225^Ac was glued to the back of the phantom filled with no water (0 cm), 4 cm, 8 cm and 12 cm of water. Measured line profiles (after background subtraction) are shown as blue dots, while corresponding simulated line profiles are shown as red lines.

## Comparison of CNR and RC for non-PVC and PVC phantom images

Table A1: RC and CNR for no PVC, RL and IY for all spheres in the phantom. Values in parentheses indicate results obtained using isocontour segmentation. The other values were obtained using CT-based segmentation.

| Energy | Sphere  Size, [ml] | Recovery coefficients (RC), [%] | | | Contrast-to-noise ratios (CNR) | | |
| --- | --- | --- | --- | --- | --- | --- | --- |
|  |  | No PVC | RL | IY | No PVC | RL | IY |
| 440 keV | 191 | 43 | 53 | 83 | 5.9 | 6.1 | 14.7 |
|  | 100 | 35 | 43 | 83 | 4.4 | 4.7 | 14.6 |
|  | 48 | 26 (33) | 33 (42) | 77 | 2.6 (3.1) | 3.1 (4.4) | 13.1 |
| 218 keV | 191 | 42 | 53 | 82 | 5.6 | 7.4 | 14.8 |
|  | 100 | 35 | 46 | 83 | 4.3 | 5.8 | 14.8 |
|  | 48 | 29 (38) | 41 (52) | 89 | 3.1 (3.4) | 4.7 (5.4) | 15.9 |
| 78 keV | 191 | 55 | 61 | 94 | 4.9 | 4.7 | 9.5 |
|  | 100 | 52 | 59 | 102 | 4.4 | 4.5 | 10.5 |
|  | 48 | 34 (51) | 39 (56) | 77 | 2.3 (3.7) | 2.5 (4.3) | 7.4 |

## Comparison of TDSC and EWSC reconstructed images for the patient study

A patient (patient 3 from figure 8), injected with 7.8 MBq of [^225^Ac]Ac -PSMA-I&T, was imaged using HE collimator 24h post-injection. 16 projections per head were acquired. The acquisition time per projection was 210s. The scatter correction was performed using either TDSC or EWSC/no EWSC. The other reconstruction and acquisition parameters were kept identical. Figure A3 shows the 24h p.i. reconstructed images for the three energy windows. All images were post-filtered using 30mm Gaussian filter.


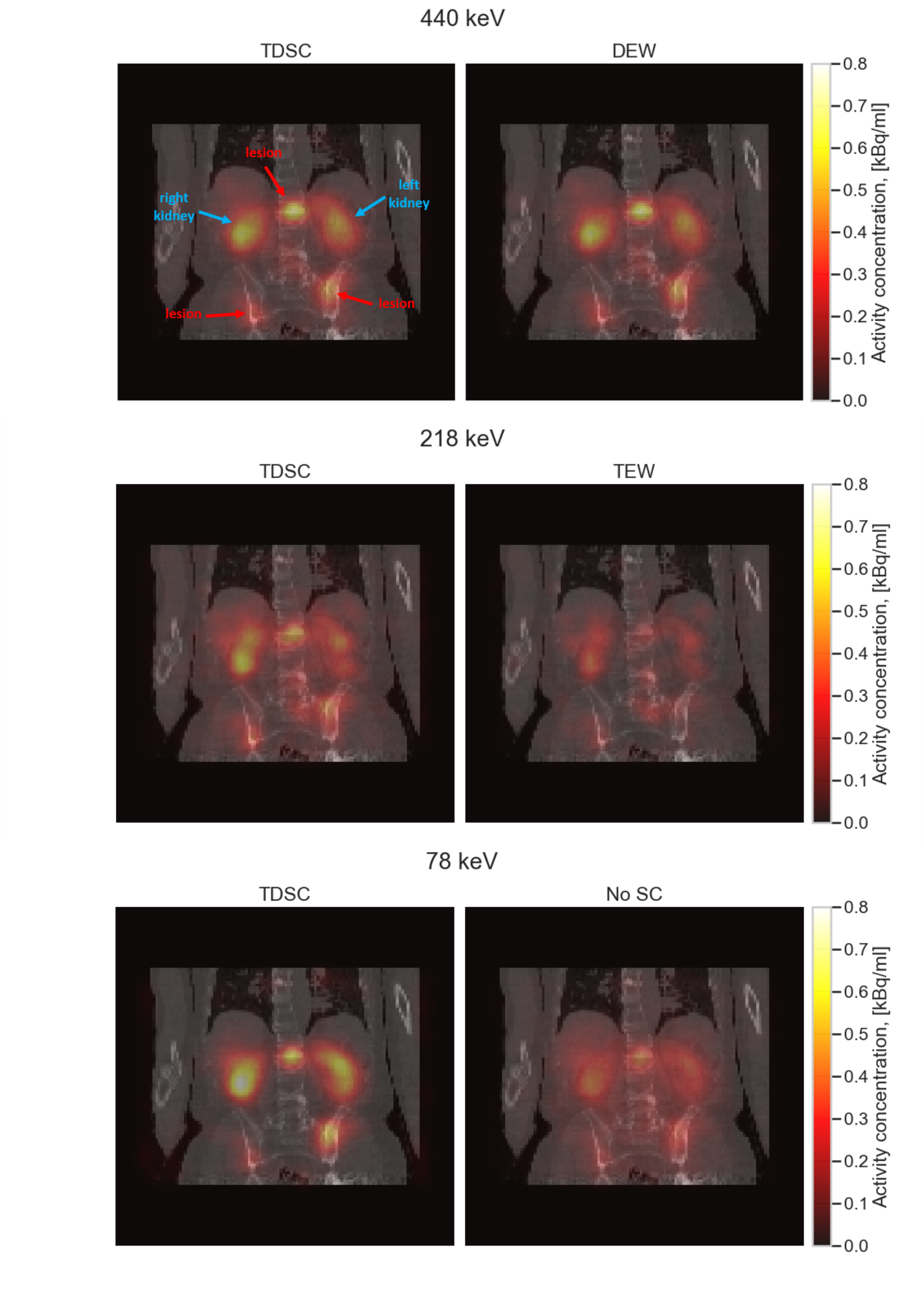


Figure A3: Comparison of TDSC and EWSC (440, 218 keV) / no SC (78 keV). For all energy windows, the same slice is shown. All images were post-filtered using 30mm Gaussian.

| **Patient** | **Kidney** | **RBE-weighted kidney absorbed dose using 440 keV peak,** $\mathbf{[}\mathbf{Gy}_{\mathbf{RBE=5}}\mathbf{]}$ | | | **RBE-weighted kidney absorbed dose using 218 keV peak,** $\mathbf{[}\mathbf{Gy}_{\mathbf{RBE=5}}\mathbf{]}$ | | | **RBE-weighted kidney absorbed dose using 78 keV peak,** $\mathbf{[}\mathbf{Gy}_{\mathbf{RBE=5}}\mathbf{]}$ | | |
| --- | --- | --- | --- | --- | --- | --- | --- | --- | --- | --- |
|  |  | **No PVC** | **IY** | **% change** | **No PVC** | **IY** | **% change** | **No PVC** | **IY** | **% change** |
| 1 | Left | 1.54 | 3.90 | 153 | 1.33 | 3.27 | 146 | 1.77 | 4.75 | 168 |
|  | Right | 1.78 | 4.41 | 148 | 1.59 | 3.84 | 142 | 2.13 | 4.88 | 129 |
| 2 | Left | 1.03 | 2.23 | 117 | 0.97 | 2.14 | 121 | 1.16 | 2.41 | 108 |
|  | Right | 1.43 | 3.89 | 172 | 1.27 | 3.48 | 174 | 1.27 | 3.20 | 152 |
| 3 | Left | 4.07 | 6.39 | 57 | 2.72 | 5.86 | 115 | 2.48 | 5.42 | 119 |
|  | Right | 2.49 | 7.28 | 192 | 2.14 | 5.40 | 152 | 3.36 | 7.65 | 128 |
| 4 | Left | 0.96 | 3.40 | 252 | 0.86 | 2.48 | 188 | 0.89 | 2.78 | 212 |
|  | Right | 0.96 | 3.11 | 224 | 0.91 | 3.36 | 269 | 1.13 | 3.87 | 242 |
| 5 | Left | 1.29 | 4.06 | 215 | 1.31 | 2.67 | 104 | 1.59 | 2.79 | 75 |
|  | Right | 1.14 | 3.36 | 195 | 0.86 | 2.26 | 163 | 1.27 | 2.92 | 130 |
| Mean ± std | | 1.67 ± 0.9 | 4.20 ± 1.4 | 172 ± 54 | 1.40 ± 0.6 | 3.48 ± 1.2 | 157 ± 45 | 1.70 ± 0.7 | 4.07 ± 1.5 | 146 ± 47 |

## Comparison of RBE-weighted kidney and lesion absorbed doses for non-PVC and PVC SPECT images

Table A2: RBE-weighted kidney absorbed doses for the patient cohort, calculated using IY PVC and non-PVC SPECT images, are presented for the three peaks. The table includes percentage changes between the two methods for each kidney and the averaged percentage changes (mean values ± standard deviations (std)) across all kidneys for each energy window.

| **Patient** | **Kidney** | **RBE-weighted kidney absorbed dose using 440 keV peak,** $\mathbf{[}\mathbf{Gy}_{\mathbf{RBE=5}}\mathbf{]}$ | | | **RBE-weighted kidney absorbed dose using 218 keV peak,** $\mathbf{[}\mathbf{Gy}_{\mathbf{RBE=5}}\mathbf{]}$ | | | **RBE-weighted kidney absorbed dose using 78 keV peak,** $\mathbf{[}\mathbf{Gy}_{\mathbf{RBE=5}}\mathbf{]}$ | | |
| --- | --- | --- | --- | --- | --- | --- | --- | --- | --- | --- |
|  |  | **No PVC** | **RL** | **% change** | **No PVC** | **RL** | **% change** | **No PVC** | **RL** | **% change** |
| 1 | Left | 1.54 | 1.79 | 16 | 1.33 | 1.49 | 12 | 1.77 | 2.19 | 24 |
|  | Right | 1.78 | 2.06 | 16 | 1.59 | 1.83 | 15 | 2.13 | 2.56 | 20 |
| 2 | Left | 1.03 | 1.37 | 33 | 0.97 | 1.24 | 28 | 1.16 | 1.61 | 39 |
|  | Right | 1.43 | 1.55 | 8 | 1.27 | 1.51 | 19 | 1.27 | 1.46 | 15 |
| 3 | Left | 4.07 | 3.88 | -5 | 2.72 | 2.96 | 9 | 2.48 | 2.94 | 19 |
|  | Right | 2.49 | 3.40 | 37 | 2.14 | 2.63 | 23 | 3.36 | 4.57 | 36 |
| 4 | Left | 0.96 | 1.16 | 21 | 0.86 | 1.02 | 19 | 0.89 | 1.12 | 26 |
|  | Right | 0.96 | 1.22 | 27 | 0.91 | 1.07 | 18 | 1.13 | 1.38 | 22 |
| 5 | Left | 1.29 | 1.46 | 13 | 1.31 | 1.39 | 6 | 1.59 | 1.59 | 0 |
|  | Right | 1.14 | 1.37 | 20 | 0.86 | 0.90 | 5 | 1.27 | 1.54 | 21 |
| Mean ± std | | 1.67 ± 0.9 | 1.93 ± 0.9 | 19 ± 11 | 1.40 ± 0.6 | 1.60 ± 0.7 | 15 ± 7 | 1.70 ± 0.7 | 2.10 ± 1.0 | 22 ± 10 |

Table A3: RBE-weighted kidney absorbed doses for the patient cohort, calculated using RL PVC and non-PVC SPECT images, are presented for the three peaks. The table includes percentage changes between the two methods for each kidney and the averaged percentage changes (mean values ± standard deviations (std)) across all kidneys for each energy window.

| **Patient** | **Lesion** | **RBE-weighted lesion absorbed dose using 440 keV peak,** $\mathbf{[}\mathbf{Gy}_{\mathbf{RBE=5}}\mathbf{]}$ | | | **RBE-weighted lesion absorbed dose using 218 keV peak,** $\mathbf{[}\mathbf{Gy}_{\mathbf{RBE=5}}\mathbf{]}$ | | | **RBE-weighted lesion absorbed dose using 78 keV peak,** $\mathbf{[}\mathbf{Gy}_{\mathbf{RBE=5}}\mathbf{]}$ | | |
| --- | --- | --- | --- | --- | --- | --- | --- | --- | --- | --- |
|  |  | **No PVC** | **RL** | **% change** | **No PVC** | **RL** | **% change** | **No PVC** | **RL** | **% change** |
| 2 | 1 | 5.18 | 7.18 | 39 | 5.44 | 7.00 | 29 | 5.90 | 6.79 | 15 |
|  | 2 | 2.11 | 2.79 | 32 | 2.17 | 2.97 | 37 | 2.36 | 3.25 | 38 |
| 3 | 1 | 2.97 | 4.12 | 39 | 3.36 | 4.34 | 29 | 2.41 | 3.36 | 39 |
|  | 2 | 3.34 | 4.24 | 27 | 2.37 | 3.14 | 32 | 2.73 | 3.37 | 23 |
|  | 3 | 2.75 | 3.79 | 38 | 3.14 | 3.95 | 26 | 1.74 | 2.24 | 29 |
| 4 | 1 | 2.75 | 3.43 | 25 | 2.26 | 3.20 | 42 | 3.56 | 4.18 | 17 |
|  | 2 | 2.52 | 3.21 | 27 | 2.08 | 2.46 | 18 | 3.24 | 3.51 | 8 |
| 5 | 1 | 1.94 | 2.79 | 44 | 1.70 | 2.32 | 36 | 1.14 | 1.40 | 23 |
|  | 2 | 2.15 | 3.01 | 40 | 2.47 | 2.83 | 15 | 3.76 | 4.29 | 14 |
| Mean ± std | | 2.86 ± 0.9 | 3.84 ± 1.3 | 34 ± 6 | 2.78 ± 1.1 | 3.58 ± 1.4 | 29 ± 8 | 2.98 ± 1.3 | 3.60 ± 1.4 | 23 ± 10 |

Table A4: RBE-weighted lesion absorbed doses for the patient cohort, calculated using RL PVC and non-PVC SPECT images, are presented for the three peaks. The table includes percentage changes between the two methods for each lesion and the averaged percentage changes (mean values ± standard deviations (std)) across all lesions for each energy window.
